# A Pilot Study Validating Video-Based Training on Pre-Hospital Stroke Recognition

**Published:** 2019-01-17

**Authors:** Aliza Brown, Sanjeeva Onteddu, Rohan Sharma, Nidhi Kapoor, Krishna Nalleballe, Appathurai Balamurugan, Sukumar Gundapaneni, Nicolas Bianchi, Robert Skinner, William Culp

**Affiliations:** 1 Department of Neurology, College of Public Health, University of Arkansas for Medical Sciences, Little Rock, AR; 2 Department of Radiology, College of Public Health, University of Arkansas for Medical Sciences, Little Rock, AR; 3 Department of Health Policy and Management, College of Public Health, University of Arkansas for Medical Sciences, Little Rock, AR; 4 Department of Chronic Disease Prevention and Control Branch, Arkansas Department of Health, Little Rock, AR; 5 Department of Neurology, Emory University, Atlanta, GA

**Keywords:** Stroke, Pre-hospital, EMS

## Abstract

**Introduction:**

Delays in recognizing stroke during pre-hospital emergency medical system (EMS) care may affect triage and transport time to an appropriate stroke ready hospital and may preclude patients from receiving time dependent treatment. All EMS transports in a large urban area in the stroke belt were evaluated for transport destinations, triage and transport time and stroke recognition following distribution ofan educational training video to local EMS services.

**Hypothesis:**

Following video training, local paramedics will improve stroke recognition and shorten triage and transport time to appropriate stroke centers of care.

**Methods:**

A training module (<10 min) containing a stroke triage scenario, instruction on the Cincinnati Prehospital Stroke Score (CPSS) and the Los Angeles Prehospital Stroke Score (LAPSS) and ‘where to transport’ stroke patients was distributed and viewed by 96 paramedics. Data was collected from February to October 2016. Stroke recognition was determined from one primary stroke center (PSC) hospital’s confirmation of EMS delivered patients (Site A). Yearly stroke recognition percentages of 44% from Site A in 2014 were used as baseline.

**Results:**

A total of 34,833 emergency 911 response transports were made with a total of 502 (1.4%) suspected strokes identified by paramedics. Median [IQR] triage and transport time for stroke transports was 33 [27­41] min. The PSC hospitals received a 5% increase in stroke transports and non-specific care facilities decreased by 7%. From 8,554 transports to site A (PSC) confirmed strokes totalled 107 transports with 139 suspected strokes by paramedics. Of these transports, 60 were correctly identified by paramedics (positive predictive value of 43%, sensitivity of 56%). By the second month following training, recognition percentages increased from baseline to 64%. At five months, percentages of correct stroke identification had dropped to 36%.

**Conclusion:**

Video based training improved stroke recognition by an additional 19%, but continual monthly or quarterly training is recommended for maintenance of increased stroke recognition.

## Introduction

Stroke is the third leading cause of mortality in the world [1]. It is also the leading cause of chronic disability in the United States (U.S) [2]. The outcome of an acute ischemic stroke depends extensively on early diagnosis and reperfusion, usually by administration of tissue plasminogen activator (tPA) [3,4]. It has been estimated that for every min that stroke is untreated, a typical patient loses about 1.9 million neurons [5]. Since the current American Stroke Association guidelines for tPA eligibility is within 4.5 hours, time is a major limiting factor for tPA administration [6,7]. Several factors can cause delay in tPA administration including delay by the patients’ families in calling 911 emergency medical services (EMS), lack of training by EMS and hospital emergency department staff, delay in obtaining radiological imaging and interpretation, and physicians’ uncertainty about administering thrombolytics [8–10].

Pre-hospital notification by emergency medical services (EMS) has been associated with improved clinical assessment and higher rates of tPA delivery with reduced door- to-needle times in the hospital [11,12]. These in turn have been found to improve stroke outcomes [13,14]. Several pre-hospital stroke assessment models have been developed for stroke screening by the EMS responders including shortened Cincinnati Prehospital Stroke Scale (CPSS), Los Angeles Motor Scale, NIH Stroke scale (NIHSS), Medic Prehospital Assessment for Code Stroke (Med PACS) and others. These assessment models have shown modest improvements in prehospital screening of stroke patients in previous studies [15–18]. We developed a video training module for pre-hospital screening and stroke recognition and distributed it to area EMS for testing.

Here we present the results over the nine-month study period to determine the training module’s efficacy.

## Methods

A review of de-identified data collected by three area Emergency Medical Systems Agencies in Central Arkansas, the Arkansas Stroke Registry (a Get-With-the-Guidelines registered program) from the Arkansas Health Department (ADH) and the University of Arkansas for Medical Sciences stroke program (Site A) was conducted. All data encompasses a review of de-identified transport data and hospital stroke confirmations from February 1, 2016 to October 31, 2016. The study was approved by the local Institutional Review Board as not human subject research.

A video utilizing professional stroke actors, paramedics and an ambulance was made to simulate a real event scenario of a 911 call for stroke. During the call and throughout the simulations of examination, a stroke educator registered nurse discussed the need to decrease time to delivery and treatment, precise methods for triage and care provided in the ambulance. Methodology for both the Los Angeles Pre-Hospital Stroke Screen (LAPSS) and the Cincinnati Pre-Hospital Stroke Screen (CPSS) were presented and discussed. Also detailed during the video was the need for early notification to alert the hospital emergency department for arrival. A directional delivery map was presented during the video illustrating the location and names of the test county’s primary stroke centers (PSC) and the one acute stroke ready hospital (ASRH). There were no comments made on the use of lights and sirens (L&S) during transport. The ADH disseminated the video link and a survey. An incentive gift card was offered to encourage video training and was given after completing an opinion survey regarding the video training.

To measure the impact of the video training on correct paramedic identification and management of ‘suspected’ strokes all transport data during the study period was collected for nine months following video release and survey return and then compared to pre-video release data that was collected a year prior to determine baseline stroke recognition rates and destination for stroke suspect transports according to hospital qualification in stroke care (non-specified care facilities (NSCF), ASRH or PSC) during the same months.

To determine the number of ‘positive’ and ‘missed’ strokes by the paramedics, all 911 ground response EMS transports delivering to one PSC (Site A) were matched against hospital ED records of strokes. Transport data used to match hospital ED cases of stroke included de-identified EMS information such as date, ER ‘seen time’, patient race, gender and age. Paramedic experience was taken into account as Years of Service (YOS) which was collected and categorized into 5 groups: <1 year, 1 to 5 years, 5 to 10 years, 10 to 15 years and over 15 years of experience. The de­identified paramedic variables of years of service and reported suspected cases of stroke were evaluated based on correct and incorrect recognition.

The paramedic’s baseline percentage of correct stroke identification was determined in a previous study which indicated that positive stroke recognition percentages were 44%. This percentage was used as a comparison for the subsequent months of positive percentages of stroke recognition.

Triage time and transport time from the scene to hospital destination were obtained for all paramedic suspected stroke transports during the study period. Both time epochs were calculated as one ‘transport time’ in min [IQR] and compared by distance travelled and travel mode (L&S or none) during the study period.

We calculated diagnostic sensitivity, specificity, accuracy and related measures across the different groups of paramedics YOS and overall sensitivity across time before and after video training.

## Results

### Video Training:

A total of 96 paramedics underwent the training with a 100% rate of reporting compliance. A total of 70 paramedics were from the test county with another 26 from surrounding areas. The survey results indicated that 9% of the paramedics had not previously performed the CPSS and 4% had not performed the LAPSS. Ninety-five percent of the paramedics thought the video would be helpful in demonstrating the use of both tests.

There were 34,833 EMS transports in the Test County during the study period (excluding 373 interfacility transports) out of which 502 (1.4%) were suspected strokes. These ‘stroke suspects’ were either transported to the ASRH, NSCF, or to area PSCs at n=5 (1%), =142 (28%) and =355 (71%), respectively. The PSC hospitals received a 5% increase in stroke transports and the NSCFs decreased by 7%. Site A received a representative sample of 139 of suspected cases out of 8,554 total EMS transports (1.6%) and took care of approximately 107 stroke patients.

Sixty paramedics transported suspected stroke patients to Site A during the study period. Their YOS are presented in Table 1 and is comparable to the paramedics YOS for all stroke transports. The paramedics with 5 to 10 YOS delivered the majority of stroke suspect transports at 41.7%. Paramedics YOS for hospital verified stroke transports to Site A during the study period are presented in Table 2.

**Figure 1:**
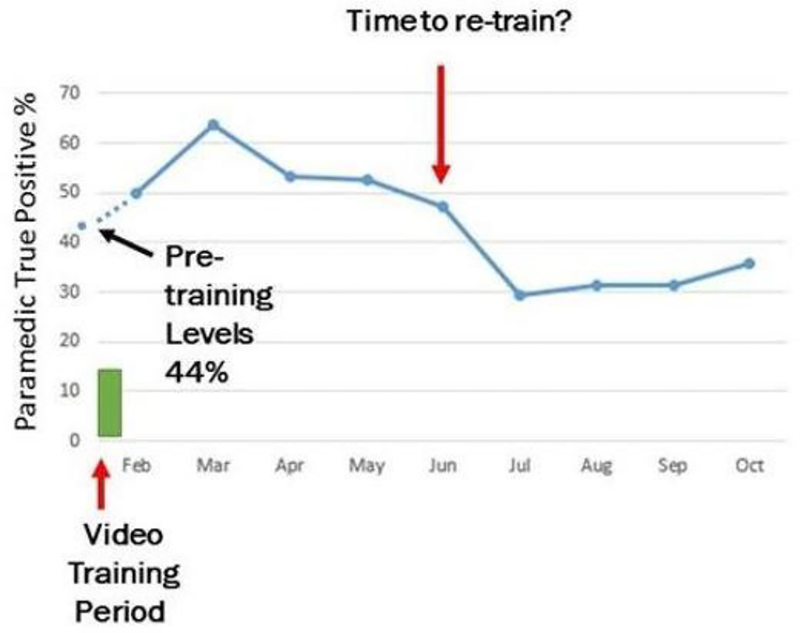
Monthly percentages of pre-hospital stroke recognition by paramedics. Pre- training recognition rates of 44% increased by 19% in the second month following training. By the fifth month (July) the rates were declining to pre-training levels.

The paramedics correctly identified stroke transports (Table 2) with a sensitivity of 56.1% [IQR 46.2–65.7] and a positive predictive value (PPV) was 43.1% [IQR36.6–50], however their diagnostic accuracy was 98.5% [IQR 98.3–98.8] due to the low prevalence of the disease among all transports. The rate of true positive recognition by the month following video dissemination is shown in Figure 1. Recognition increased by 5% during the month of training and an additional 14% by the second month but then gradually declined to baseline or lower at 35.7% within four months.

Overall combined triage and transport time for all stroke suspects was 33 min [IQR 27–41]. Data on median triage and transport time by vehicle territory is shown in Table 3.

**Figure 2:**
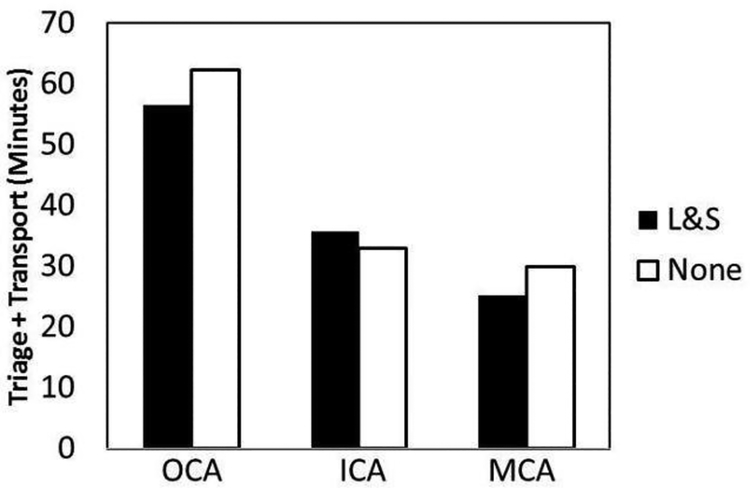
Combining median triage and transport time and vehicle territory to hospital destination with the transport mode variable (Lights and Sirens [L&S]). Vehicles utilizing lights and sirens (L&S) decreased median triage and transport time to area hospitals in the study test county compared to no lights and sirens (None) from transports originating from outside county area (OCA) and in the metro county area (MCA) territories. The in-county area (ICA) stroke transports did not improve median triage and transport time using L&S. Median data represented. Black bars represent L&S and None are shown by the white bars.

The data indicates that transports inside the county area and metro areas had similar median times to destination hospitals. Some stroke transports used lights and sirens (L&S) during commute and influenced transport time as shown in Figure 2.

Vehicles originating from outside county areas (OCA) that used L&S had shorter median delivery times *vs.* no L&S (None) transports (56.6 [IQR 36.7–58] *vs.* 62.4 [IQR 47–69] min).

Transports within the county area (ICA) did not decrease delivery time with L&S *vs.* None at 35.7 [IQR27.7–44] *vs.* 32.9 [IQR 29–43], respectively.

Those transports using L&S in the metro county area (MCA) had an improvement *vs.* None at 25.3 [IQR 20.2– 32] *vs.* 30 [IQR 25–35] min, respectively.

## Discussion

Pre-hospital stroke assessment and notifications have been known to decrease the door to needle time, increase the rate of tPA delivery, and improve stroke outcomes [11–14]. Previous studies have found the diverse sensitivity of prehospital stroke assessment increase from 44% to as high as 91% [19–21]. Such varied results are due to differences in assessment scales, state population and location, urban versus rural settings, training level of paramedics, volume of strokes seen by paramedics and other factors such as trained emergency medical dispatchers (EMD).

Following stroke recognition training in this study, the dramatic 19% increase from baseline in sensitivity (Figure 1) was seen within two months. Wojner-Alexandrov et al. [20] also observed similar results in their study. The video training offered in this study has the potential to benefit the entire state if adapted for county specific ASRH hospital locations considering the large telestroke network within the state [22].

The training may have also helped the paramedics in directing potential stroke patients to PSCs, which are better equipped at handling stroke cases. Although rates of endovascular interventions on these patients were not studied, this might be important to analyze in future studies. Rates of endovascular intervention could be compared to large vessel occlusion detection scores learned from video-based training in prehospital screening and triage. The use of L&S added an additional variable on triage and transport time (Figure 2). The median transport time for the suspected strokes indicated an improvement in time reduction when L&S were used by paramedics in the OCA territory although its use was not discussed in the video. Triage and transport time in the metro county study area (MCA) compared to rural in county areas (ICA) were consistent (Table 3) until the transport modes using L&S were applied (Figure 2). The data here suggests the use of L&S in heavy city traffic (MCA) and on the longer highway commutes (OCA) entering the county area (Figure 2). Although more data and more variables need to be analyzed to draw conclusions about the use of L&S in stroke transport in our region.

Falling stroke recognition rates (Table 2, Figure 1), suggests the need for retraining at regular intervals. Further decreases in stroke recognition rate may have included potential seasonal effects. However, our video training provides a cost effective and easily accessible retraining instrument for this purpose. Similar to this study, re-training after 5–6 months has been utilized in other studies examining training methods for pre­hospital stroke recognition, which recommended retraining at 3 to 6 months [23–25].

Since the overall percentage of stroke patients assessed by paramedics in our study was very low (1.4%), the paramedics may be less adept at recognizing stroke cases as compared to areas yielding a higher stroke volume [23]. The video training did improve paramedic stroke recognition rates by 19% and may continue to improve with retraining.

## Figures and Tables

**Table 1:** Number of Paramedic’s by Years of Service (YOS).

Criteria	Delivering to Site A for Stroke Recognition	Delivering to all hospitals
YOS	n (%)	n (%)
<1 year	7 (11.7)	9 (9.1)
1<YOS<5	14 (23.3)	26 (26.5)
5<YOS<10	25 (41.7)	36 (36.7)
10<YOS<15	12 (20.0)	19 (19.3)
YOS>15	2 (3.3)	8 (8.1)

**Table 2:** Number of stroke recognition transports by paramedics delivering to Site A; (Total transports=8.554).

Years of Service (YOS)	Both Medic and hospital identified as stroke (n)		Medic did not identify stroke, but hospital identified stroke (n)		Medic identified stroke, but hospital did not identify stroke (n)		Totals	
	True Positive* (56%)		False Negative**		False Positive*			
YOS<=1	2	3.30%	3	6.40%	6	7.60%	11	5.90%
1<YOS<5	9	15.00%	15	31.90%	19	24.00%	43	23.10%
5<YOS<10	37	61.70%	17	36.20%	36	45.60%	90	43.40%
10<YOS<15	11	18.30%	11	23.40%	13	16.50%	35	18.80%
YOS>15	1	1.70%	1	2.10%	5	6.30%	7	3.80%
Totals	60		47		79		186	

* Out of 139 (60+79) medic identified strokes, 60 were positive as validated by the hospital (Site A). True negatives=8368.

** The paramedics missed 47 strokes and were identified in the total transports with other impression codes (false negatives).

**Table 3:** Median triage plus transport time of paramedic suspected strokes.

Vehicle Territory*	N	Median Time (min)	[Q1–Q3]
OCA	11	58.4	[44.1–65.7]
ICA	23	33.1	[29.3–43.7]
MCA	105	29	[23.7–34.5]

* OCA: Outside County Area; ICA: Inside County Area; MCA=In the Metro County Area
